# Crystal structure of (*E*)-1-(4-chloro­phen­yl)ethanone *O*-de­hydro­abietyloxime

**DOI:** 10.1107/S1600536814015888

**Published:** 2014-08-01

**Authors:** Jian-Qiang Zheng, Yan-Jie Cui, Xiao-Ping Rao

**Affiliations:** a Institute of Chemical Industry of Forest Products, Chinese Academy of Forestry, Key Lab. of Biomass Energy and Material, Jiangsu Province, National Engineering Lab. for Biomass Chemical Utilization, Key and Lab. on Forest Chemical Engineering, SFA, Nanjing, 210042, People’s Republic of China

**Keywords:** crystal structure, dehydroabietic acid derivative, oxime

## Abstract

The title compound, C_28_H_34_ClNO_2_ {systematic name: (*E*)-1-(4-chloro­phen­yl)ethanone *O*-[(1*R*,4a*S*,10a*R*)-7-isopropyl-1,4a-di­methyl-1,2,3,4,4a,9,10,10a-octa­hydro­phenanthrene-1-carbonyl]oxime}, was synthesized from de­hydro­abietic acid. In the de­hydro­abietyl moiety, the central and terminal cyclo­hexane rings display chair and half-chair conformations, respectively, and a *trans*-ring junction. The C=N bond is in an *E* conformation and the C—O—N=C torsion angle is 148.1 (5)°. No directional inter­actions except van der Waals contacts occur in the crystal structure.

## Related literature   

For the biological activity of de­hydro­abietic acid derivatives, see: Cui *et al.* (2013 ▶); Rao *et al.* (2008 ▶); Sepulveda *et al.* (2005 ▶). For the crystal structures of de­hydro­abietic acid derivatives, see: Rao *et al.* (2006 ▶, 2009 ▶).
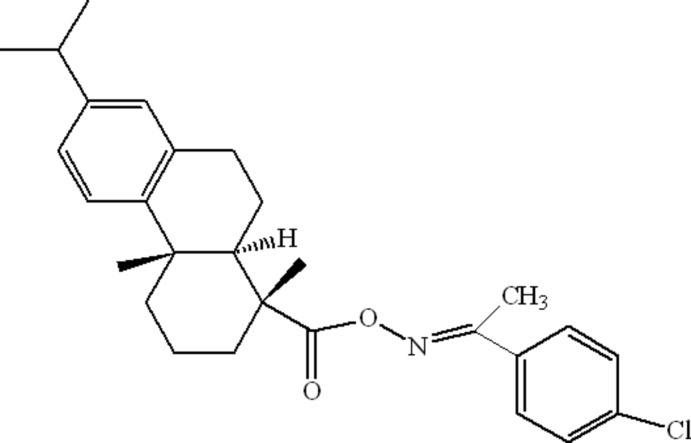


## Experimental   

### Crystal data  

C_28_H_34_ClNO_2_
*M* *_r_* = 452.01
Orthorhombic, 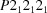

*a* = 28.804 (6) Å
*b* = 6.1760 (12) Å
*c* = 13.922 (3) Å
*V* = 2476.6 (9) Å^3^
*Z* = 4
Mo *K*α radiation
μ = 0.18 mm^−1^
*T* = 293 K
0.30 × 0.20 × 0.10 mm

### Data collection  

Enraf–Nonius CAD-4 diffractometer
Absorption correction: ψ scan (North *et al.*, 1968 ▶) *T*_min_ = 0.948, *T*_max_ = 0.982
5186 measured reflections
4554 independent reflections
2566 reflections with *I* > 2σ(*I*)
*R*_int_ = 0.099
3 standard reflections every 200 reflections intensity decay: 1%

### Refinement  

*R*[*F* ^2^ > 2σ(*F* ^2^)] = 0.069
*wR*(*F* ^2^) = 0.205
*S* = 1.00
4554 reflections
289 parameters
H-atom parameters constrained
Δρ_max_ = 0.18 e Å^−3^
Δρ_min_ = −0.23 e Å^−3^
Absolute structure: Flack (1983 ▶), 1911 Friedel pairs
Absolute structure parameter: 0.05 (16)

### 

Data collection: *CAD-4 Software* (Enraf–Nonius, 1989 ▶); cell refinement: *CAD-4 Software* data reduction: *XCAD4* (Harms & Wocadlo, 1995 ▶); program(s) used to solve structure: *SHELXS97* (Sheldrick, 2008 ▶); program(s) used to refine structure: *SHELXL97* (Sheldrick, 2008 ▶); molecular graphics: *SHELXTL* (Sheldrick, 2008 ▶); software used to prepare material for publication: *SHELXTL*.

## Supplementary Material

Crystal structure: contains datablock(s) I, global. DOI: 10.1107/S1600536814015888/hb7249sup1.cif

Structure factors: contains datablock(s) I. DOI: 10.1107/S1600536814015888/hb7249Isup2.hkl

**Fig. 1.**
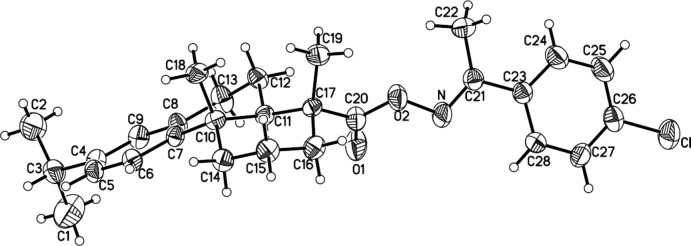
Molecular structure of the title compound, with displacement ellipsoids drawn at the 30% probability level.

. DOI: 10.1107/S1600536814015888/hb7249fig1.tif
Mol­ecular structure of the title compound, with displacement ellipsoids drawn at the 30% probability level.

CCDC reference: 1012686

Additional supporting information:  crystallographic information; 3D view; checkCIF report

## supplementary crystallographic information

### S1. Experimental

A solution of dehydroabietyl chloride in 15 ml CH_2_Cl_2_ solution were added dropwise to a solution of 60 mmol oxime and 60 mmol triethylamine in 40 ml CH_2_Cl_2_ solution within 30 min between the temperature 0–5 °C. The reaction mixture was allowed to stand at room temperature over 2 h and washed with water, then dried with anhydrous MgSO_4_. The residue were purified by silica gel chromatography.

### S2. Refinement

H atoms were positioned geometrically and refined as riding atoms, with C—H = 0.96 Å and *U*_iso_(H) = 1.5*U*_eq_(C) for methyl H atoms, and C—H = 0.97–0.98 Å and *U*_iso_(H) = 1.2*U*_eq_(C,*N*,H) for all other H atoms. Methyl groups were refined in orientation (AFIX 137 of program *SHELXL97*.

### Crystal data

**Table:**

C_28_H_34_ClNO_2_	*F*(000) = 968
*M**_r_* = 452.01	*D*_x_ = 1.212 Mg m^−^^3^
Orthorhombic, *P*2_1_2_1_2_1_	Mo *K*α radiation, λ = 0.71073 Å
Hall symbol: P 2ac 2ab	Cell parameters from 25 reflections
*a* = 28.804 (6) Å	θ = 9–13°
*b* = 6.1760 (12) Å	µ = 0.18 mm^−^^1^
*c* = 13.922 (3) Å	*T* = 293 K
*V* = 2476.6 (9) Å^3^	Block, colourless
*Z* = 4	0.30 × 0.20 × 0.10 mm

### Data collection

**Table:**

Enraf–Nonius CAD-4 diffractometer	2566 reflections with *I* > 2σ(*I*)
Radiation source: fine-focus sealed tube	*R*_int_ = 0.099
Graphite monochromator	θ_max_ = 25.4°, θ_min_ = 1.4°
ω/2θ scans	*h* = −34→34
Absorption correction: ψ scan (North *et al.*, 1968)	*k* = 0→7
*T*_min_ = 0.948, *T*_max_ = 0.982	*l* = 0→16
5186 measured reflections	3 standard reflections every 200 reflections
4554 independent reflections	intensity decay: 1%

### Refinement

**Table:**

Refinement on *F*^2^	Secondary atom site location: difference Fourier map
Least-squares matrix: full	Hydrogen site location: inferred from neighbouring sites
*R*[*F*^2^ > 2σ(*F*^2^)] = 0.069	H-atom parameters constrained
*wR*(*F*^2^) = 0.205	*w* = 1/[σ^2^(*F*_o_^2^) + (0.1*P*)^2^ + 0.3*P*] where *P* = (*F*_o_^2^ + 2*F*_c_^2^)/3
*S* = 1.00	(Δ/σ)_max_ < 0.001
4554 reflections	Δρ_max_ = 0.18 e Å^−^^3^
289 parameters	Δρ_min_ = −0.23 e Å^−^^3^
0 restraints	Absolute structure: Flack (1983), 1911 Friedel pairs
Primary atom site location: structure-invariant direct methods	Absolute structure parameter: 0.05 (16)

### Special details

**Table:**

Geometry. All e.s.d.'s (except the e.s.d. in the dihedral angle between two l.s. planes) are estimated using the full covariance matrix. The cell e.s.d.'s are taken into account individually in the estimation of e.s.d.'s in distances, angles and torsion angles; correlations between e.s.d.'s in cell parameters are only used when they are defined by crystal symmetry. An approximate (isotropic) treatment of cell e.s.d.'s is used for estimating e.s.d.'s involving l.s. planes.
Refinement. Refinement of *F*^2^ against ALL reflections. The weighted *R*-factor *wR* and goodness of fit *S* are based on *F*^2^, conventional *R*-factors *R* are based on *F*, with *F* set to zero for negative *F*^2^. The threshold expression of *F*^2^ > σ(*F*^2^) is used only for calculating *R*-factors(gt) *etc*. and is not relevant to the choice of reflections for refinement. *R*-factors based on *F*^2^ are statistically about twice as large as those based on *F*, and *R*- factors based on ALL data will be even larger.

### Fractional atomic coordinates and isotropic or equivalent isotropic displacement parameters (Å^2^)

**Table:**

	*x*	*y*	*z*	*U*_iso_*/*U*_eq_	
Cl	0.59358 (6)	0.1759 (3)	0.58245 (11)	0.0903 (6)	
N	0.41858 (16)	0.1075 (8)	0.2777 (4)	0.0786 (15)	
O1	0.34454 (14)	0.3392 (7)	0.2493 (3)	0.0917 (14)	
C1	0.0874 (3)	0.9817 (14)	0.1786 (7)	0.143 (4)	
H1A	0.0598	1.0654	0.1890	0.214*	
H1B	0.1088	1.0637	0.1403	0.214*	
H1C	0.1014	0.9479	0.2393	0.214*	
O2	0.38242 (13)	0.0305 (7)	0.2150 (3)	0.0774 (12)	
C2	0.0380 (2)	0.6446 (14)	0.1772 (5)	0.106 (2)	
H2A	0.0303	0.5175	0.1409	0.159*	
H2B	0.0109	0.7334	0.1844	0.159*	
H2C	0.0492	0.6028	0.2394	0.159*	
C3	0.07480 (18)	0.7692 (9)	0.1256 (4)	0.0707 (16)	
H3A	0.0617	0.8110	0.0634	0.085*	
C4	0.11734 (16)	0.6380 (9)	0.1050 (4)	0.0564 (13)	
C5	0.13208 (17)	0.5982 (9)	0.0124 (4)	0.0574 (13)	
H5A	0.1154	0.6568	−0.0386	0.069*	
C6	0.17069 (17)	0.4744 (8)	−0.0067 (3)	0.0527 (12)	
H6A	0.1799	0.4552	−0.0701	0.063*	
C7	0.19647 (15)	0.3765 (8)	0.0666 (3)	0.0463 (11)	
C8	0.18179 (17)	0.4124 (8)	0.1606 (3)	0.0525 (13)	
C9	0.14295 (17)	0.5418 (9)	0.1772 (4)	0.0574 (13)	
H9A	0.1338	0.5645	0.2404	0.069*	
C10	0.23686 (16)	0.2213 (8)	0.0425 (3)	0.0480 (11)	
C11	0.26936 (15)	0.2185 (8)	0.1322 (3)	0.0492 (11)	
H11A	0.2772	0.3707	0.1440	0.059*	
C12	0.24209 (17)	0.1469 (9)	0.2209 (3)	0.0560 (13)	
H12A	0.2265	0.0108	0.2079	0.067*	
H12B	0.2632	0.1252	0.2744	0.067*	
C13	0.20670 (18)	0.3184 (11)	0.2463 (3)	0.0711 (16)	
H13A	0.1839	0.2558	0.2895	0.085*	
H13B	0.2222	0.4346	0.2805	0.085*	
C14	0.26495 (17)	0.3094 (9)	−0.0430 (3)	0.0589 (13)	
H14A	0.2462	0.3009	−0.1007	0.071*	
H14B	0.2722	0.4606	−0.0317	0.071*	
C15	0.30964 (17)	0.1850 (10)	−0.0586 (3)	0.0646 (14)	
H15A	0.3024	0.0354	−0.0737	0.077*	
H15B	0.3262	0.2463	−0.1129	0.077*	
C16	0.34032 (18)	0.1924 (10)	0.0290 (3)	0.0631 (14)	
H16A	0.3496	0.3412	0.0406	0.076*	
H16B	0.3682	0.1091	0.0165	0.076*	
C17	0.31646 (17)	0.1025 (7)	0.1202 (3)	0.0487 (11)	
C18	0.21533 (19)	0.0064 (8)	0.0147 (4)	0.0611 (14)	
H18A	0.1982	−0.0505	0.0682	0.092*	
H18B	0.2394	−0.0936	−0.0029	0.092*	
H18C	0.1948	0.0272	−0.0388	0.092*	
C19	0.3132 (2)	−0.1455 (8)	0.1193 (4)	0.0704 (15)	
H19A	0.3437	−0.2060	0.1118	0.106*	
H19B	0.2939	−0.1909	0.0668	0.106*	
H19C	0.2999	−0.1948	0.1787	0.106*	
C20	0.34771 (18)	0.1739 (10)	0.2020 (4)	0.0627 (14)	
C21	0.43576 (18)	−0.0547 (9)	0.3230 (4)	0.0562 (13)	
C22	0.4184 (2)	−0.2817 (10)	0.3171 (5)	0.0831 (19)	
H22A	0.3926	−0.2881	0.2734	0.125*	
H22B	0.4085	−0.3287	0.3796	0.125*	
H22C	0.4428	−0.3745	0.2945	0.125*	
C23	0.47602 (16)	−0.0019 (9)	0.3849 (3)	0.0551 (12)	
C24	0.4921 (2)	−0.1439 (10)	0.4542 (4)	0.0766 (17)	
H24A	0.4777	−0.2780	0.4605	0.092*	
C25	0.5287 (2)	−0.0937 (11)	0.5145 (4)	0.0809 (19)	
H25A	0.5389	−0.1922	0.5604	0.097*	
C26	0.54968 (19)	0.1069 (11)	0.5048 (4)	0.0647 (15)	
C27	0.53477 (18)	0.2524 (11)	0.4368 (4)	0.0698 (16)	
H27A	0.5491	0.3868	0.4314	0.084*	
C28	0.49870 (18)	0.1988 (9)	0.3768 (4)	0.0653 (14)	
H28A	0.4891	0.2968	0.3301	0.078*	

### Atomic displacement parameters (Å^2^)

**Table:**

	*U*^11^	*U*^22^	*U*^33^	*U*^12^	*U*^13^	*U*^23^
Cl	0.0746 (9)	0.1184 (14)	0.0778 (10)	−0.0015 (10)	−0.0227 (8)	0.0008 (10)
N	0.069 (3)	0.072 (3)	0.095 (4)	−0.004 (3)	−0.039 (3)	−0.005 (3)
O1	0.080 (3)	0.081 (3)	0.113 (3)	0.019 (2)	−0.041 (3)	−0.049 (3)
C1	0.094 (5)	0.082 (5)	0.253 (11)	0.014 (5)	0.004 (6)	−0.073 (7)
O2	0.070 (2)	0.062 (2)	0.099 (3)	0.011 (2)	−0.036 (2)	−0.017 (2)
C2	0.069 (4)	0.109 (6)	0.139 (6)	0.014 (4)	0.026 (4)	0.023 (5)
C3	0.064 (3)	0.067 (4)	0.080 (4)	0.011 (3)	0.000 (3)	0.003 (3)
C4	0.050 (3)	0.062 (3)	0.057 (3)	−0.001 (3)	−0.004 (2)	−0.004 (3)
C5	0.054 (3)	0.064 (3)	0.054 (3)	0.001 (3)	−0.014 (2)	0.000 (3)
C6	0.060 (3)	0.059 (3)	0.039 (3)	0.003 (3)	−0.006 (2)	−0.005 (2)
C7	0.045 (2)	0.054 (3)	0.040 (3)	−0.001 (2)	−0.0046 (19)	−0.003 (2)
C8	0.052 (3)	0.066 (3)	0.040 (3)	0.011 (3)	−0.003 (2)	0.003 (2)
C9	0.056 (3)	0.073 (3)	0.044 (3)	0.001 (3)	0.001 (2)	−0.004 (3)
C10	0.055 (3)	0.052 (3)	0.038 (2)	−0.007 (2)	0.000 (2)	−0.002 (2)
C11	0.047 (2)	0.055 (3)	0.046 (3)	−0.003 (2)	−0.003 (2)	0.001 (2)
C12	0.062 (3)	0.059 (3)	0.046 (3)	0.005 (3)	−0.003 (2)	0.009 (2)
C13	0.071 (3)	0.099 (4)	0.044 (3)	0.021 (4)	0.006 (3)	0.009 (3)
C14	0.063 (3)	0.074 (3)	0.040 (3)	−0.002 (3)	0.001 (2)	0.005 (3)
C15	0.060 (3)	0.074 (4)	0.059 (3)	0.005 (3)	0.014 (3)	0.001 (3)
C16	0.057 (3)	0.070 (3)	0.063 (3)	−0.001 (3)	0.005 (3)	−0.007 (3)
C17	0.050 (3)	0.042 (3)	0.054 (3)	−0.006 (2)	−0.006 (2)	−0.005 (2)
C18	0.071 (3)	0.049 (3)	0.063 (3)	−0.013 (3)	−0.011 (3)	−0.008 (2)
C19	0.079 (4)	0.043 (3)	0.089 (4)	−0.002 (3)	−0.015 (3)	−0.010 (3)
C20	0.055 (3)	0.067 (4)	0.065 (3)	0.007 (3)	−0.009 (3)	−0.006 (3)
C21	0.055 (3)	0.062 (3)	0.052 (3)	0.002 (3)	0.000 (2)	0.001 (3)
C22	0.079 (4)	0.071 (4)	0.099 (5)	−0.021 (3)	−0.014 (3)	0.027 (3)
C23	0.049 (3)	0.064 (3)	0.053 (3)	0.004 (3)	0.004 (2)	0.003 (3)
C24	0.086 (4)	0.070 (4)	0.074 (4)	0.005 (4)	−0.017 (3)	0.012 (3)
C25	0.091 (5)	0.077 (4)	0.075 (4)	0.009 (4)	−0.025 (4)	0.015 (3)
C26	0.058 (3)	0.084 (4)	0.052 (3)	0.011 (3)	−0.003 (3)	0.002 (3)
C27	0.053 (3)	0.082 (4)	0.074 (4)	−0.011 (3)	−0.008 (3)	0.004 (3)
C28	0.061 (3)	0.065 (3)	0.070 (3)	−0.004 (3)	−0.008 (3)	0.020 (3)

### Geometric parameters (Å, º)

**Table:**

Cl—C26	1.717 (6)	C13—H13A	0.9700
N—C21	1.284 (7)	C13—H13B	0.9700
N—O2	1.441 (5)	C14—C15	1.515 (7)
O1—C20	1.218 (6)	C14—H14A	0.9700
C1—C3	1.549 (9)	C14—H14B	0.9700
C1—H1A	0.9600	C15—C16	1.507 (7)
C1—H1B	0.9600	C15—H15A	0.9700
C1—H1C	0.9600	C15—H15B	0.9700
O2—C20	1.348 (6)	C16—C17	1.547 (7)
C2—C3	1.493 (8)	C16—H16A	0.9700
C2—H2A	0.9600	C16—H16B	0.9700
C2—H2B	0.9600	C17—C20	1.517 (7)
C2—H2C	0.9600	C17—C19	1.535 (6)
C3—C4	1.497 (7)	C18—H18A	0.9600
C3—H3A	0.9800	C18—H18B	0.9600
C4—C5	1.379 (7)	C18—H18C	0.9600
C4—C9	1.381 (7)	C19—H19A	0.9600
C5—C6	1.376 (7)	C19—H19B	0.9600
C5—H5A	0.9300	C19—H19C	0.9600
C6—C7	1.399 (6)	C21—C23	1.481 (7)
C6—H6A	0.9300	C21—C22	1.491 (8)
C7—C8	1.393 (6)	C22—H22A	0.9600
C7—C10	1.544 (7)	C22—H22B	0.9600
C8—C9	1.394 (7)	C22—H22C	0.9600
C8—C13	1.509 (6)	C23—C24	1.383 (7)
C9—H9A	0.9300	C23—C28	1.406 (7)
C10—C18	1.515 (7)	C24—C25	1.383 (8)
C10—C14	1.538 (6)	C24—H24A	0.9300
C10—C11	1.561 (6)	C25—C26	1.385 (9)
C11—C12	1.528 (6)	C25—H25A	0.9300
C11—C17	1.543 (7)	C26—C27	1.374 (8)
C11—H11A	0.9800	C27—C28	1.374 (7)
C12—C13	1.512 (7)	C27—H27A	0.9300
C12—H12A	0.9700	C28—H28A	0.9300
C12—H12B	0.9700		
			
C21—N—O2	108.6 (5)	C10—C14—H14A	109.2
C3—C1—H1A	109.5	C15—C14—H14B	109.2
C3—C1—H1B	109.5	C10—C14—H14B	109.2
H1A—C1—H1B	109.5	H14A—C14—H14B	107.9
C3—C1—H1C	109.5	C16—C15—C14	111.5 (4)
H1A—C1—H1C	109.5	C16—C15—H15A	109.3
H1B—C1—H1C	109.5	C14—C15—H15A	109.3
C20—O2—N	113.6 (4)	C16—C15—H15B	109.3
C3—C2—H2A	109.5	C14—C15—H15B	109.3
C3—C2—H2B	109.5	H15A—C15—H15B	108.0
H2A—C2—H2B	109.5	C15—C16—C17	113.1 (4)
C3—C2—H2C	109.5	C15—C16—H16A	108.9
H2A—C2—H2C	109.5	C17—C16—H16A	108.9
H2B—C2—H2C	109.5	C15—C16—H16B	108.9
C2—C3—C4	113.2 (5)	C17—C16—H16B	108.9
C2—C3—C1	112.0 (6)	H16A—C16—H16B	107.8
C4—C3—C1	111.0 (5)	C20—C17—C19	109.5 (4)
C2—C3—H3A	106.7	C20—C17—C11	107.8 (4)
C4—C3—H3A	106.7	C19—C17—C11	114.2 (4)
C1—C3—H3A	106.7	C20—C17—C16	104.4 (4)
C5—C4—C9	116.1 (5)	C19—C17—C16	112.2 (4)
C5—C4—C3	121.9 (5)	C11—C17—C16	108.2 (4)
C9—C4—C3	122.0 (5)	C10—C18—H18A	109.5
C6—C5—C4	121.9 (5)	C10—C18—H18B	109.5
C6—C5—H5A	119.0	H18A—C18—H18B	109.5
C4—C5—H5A	119.0	C10—C18—H18C	109.5
C5—C6—C7	121.9 (5)	H18A—C18—H18C	109.5
C5—C6—H6A	119.0	H18B—C18—H18C	109.5
C7—C6—H6A	119.0	C17—C19—H19A	109.5
C8—C7—C6	117.1 (4)	C17—C19—H19B	109.5
C8—C7—C10	122.1 (4)	H19A—C19—H19B	109.5
C6—C7—C10	120.7 (4)	C17—C19—H19C	109.5
C7—C8—C9	119.4 (4)	H19A—C19—H19C	109.5
C7—C8—C13	122.5 (4)	H19B—C19—H19C	109.5
C9—C8—C13	118.1 (4)	O1—C20—O2	122.3 (5)
C4—C9—C8	123.7 (5)	O1—C20—C17	127.2 (5)
C4—C9—H9A	118.2	O2—C20—C17	110.4 (5)
C8—C9—H9A	118.2	N—C21—C23	114.6 (5)
C18—C10—C14	109.1 (4)	N—C21—C22	125.3 (5)
C18—C10—C7	106.9 (4)	C23—C21—C22	120.2 (5)
C14—C10—C7	110.2 (4)	C21—C22—H22A	109.5
C18—C10—C11	116.1 (4)	C21—C22—H22B	109.5
C14—C10—C11	107.9 (4)	H22A—C22—H22B	109.5
C7—C10—C11	106.5 (3)	C21—C22—H22C	109.5
C12—C11—C17	113.9 (4)	H22A—C22—H22C	109.5
C12—C11—C10	109.9 (4)	H22B—C22—H22C	109.5
C17—C11—C10	116.5 (4)	C24—C23—C28	117.4 (5)
C12—C11—H11A	105.1	C24—C23—C21	121.9 (5)
C17—C11—H11A	105.1	C28—C23—C21	120.8 (5)
C10—C11—H11A	105.1	C25—C24—C23	122.4 (6)
C13—C12—C11	109.5 (4)	C25—C24—H24A	118.8
C13—C12—H12A	109.8	C23—C24—H24A	118.8
C11—C12—H12A	109.8	C24—C25—C26	118.3 (6)
C13—C12—H12B	109.8	C24—C25—H25A	120.8
C11—C12—H12B	109.8	C26—C25—H25A	120.8
H12A—C12—H12B	108.2	C27—C26—C25	121.0 (5)
C8—C13—C12	113.9 (4)	C27—C26—Cl	120.1 (5)
C8—C13—H13A	108.8	C25—C26—Cl	118.8 (5)
C12—C13—H13A	108.8	C28—C27—C26	119.9 (6)
C8—C13—H13B	108.8	C28—C27—H27A	120.1
C12—C13—H13B	108.8	C26—C27—H27A	120.1
H13A—C13—H13B	107.7	C27—C28—C23	121.0 (5)
C15—C14—C10	112.2 (4)	C27—C28—H28A	119.5
C15—C14—H14A	109.2	C23—C28—H28A	119.5
			
C21—N—O2—C20	148.1 (5)	C11—C10—C14—C15	54.1 (5)
C2—C3—C4—C5	118.1 (6)	C10—C14—C15—C16	−58.8 (6)
C1—C3—C4—C5	−115.0 (7)	C14—C15—C16—C17	57.7 (6)
C2—C3—C4—C9	−59.0 (7)	C12—C11—C17—C20	−67.0 (5)
C1—C3—C4—C9	68.0 (8)	C10—C11—C17—C20	163.4 (4)
C9—C4—C5—C6	−1.6 (7)	C12—C11—C17—C19	54.9 (6)
C3—C4—C5—C6	−178.8 (5)	C10—C11—C17—C19	−74.7 (5)
C4—C5—C6—C7	1.9 (8)	C12—C11—C17—C16	−179.3 (4)
C5—C6—C7—C8	−1.0 (7)	C10—C11—C17—C16	51.1 (5)
C5—C6—C7—C10	174.3 (4)	C15—C16—C17—C20	−166.7 (5)
C6—C7—C8—C9	−0.1 (7)	C15—C16—C17—C19	74.9 (6)
C10—C7—C8—C9	−175.3 (4)	C15—C16—C17—C11	−52.1 (6)
C6—C7—C8—C13	−179.4 (5)	N—O2—C20—O1	−9.4 (8)
C10—C7—C8—C13	5.4 (8)	N—O2—C20—C17	168.2 (4)
C5—C4—C9—C8	0.5 (8)	C19—C17—C20—O1	−147.7 (6)
C3—C4—C9—C8	177.7 (5)	C11—C17—C20—O1	−23.0 (8)
C7—C8—C9—C4	0.3 (8)	C16—C17—C20—O1	91.9 (7)
C13—C8—C9—C4	179.6 (5)	C19—C17—C20—O2	34.8 (6)
C8—C7—C10—C18	97.1 (5)	C11—C17—C20—O2	159.5 (4)
C6—C7—C10—C18	−77.9 (5)	C16—C17—C20—O2	−85.6 (5)
C8—C7—C10—C14	−144.4 (5)	O2—N—C21—C23	175.4 (4)
C6—C7—C10—C14	40.6 (6)	O2—N—C21—C22	−4.5 (8)
C8—C7—C10—C11	−27.6 (6)	N—C21—C23—C24	165.3 (5)
C6—C7—C10—C11	157.4 (4)	C22—C21—C23—C24	−14.8 (8)
C18—C10—C11—C12	−61.1 (5)	N—C21—C23—C28	−13.0 (7)
C14—C10—C11—C12	176.1 (4)	C22—C21—C23—C28	167.0 (5)
C7—C10—C11—C12	57.8 (5)	C28—C23—C24—C25	0.4 (8)
C18—C10—C11—C17	70.3 (5)	C21—C23—C24—C25	−177.9 (5)
C14—C10—C11—C17	−52.5 (5)	C23—C24—C25—C26	0.3 (9)
C7—C10—C11—C17	−170.8 (4)	C24—C25—C26—C27	−0.3 (9)
C17—C11—C12—C13	159.6 (4)	C24—C25—C26—Cl	177.3 (5)
C10—C11—C12—C13	−67.6 (5)	C25—C26—C27—C28	−0.5 (9)
C7—C8—C13—C12	−12.1 (8)	Cl—C26—C27—C28	−178.0 (4)
C9—C8—C13—C12	168.6 (5)	C26—C27—C28—C23	1.2 (8)
C11—C12—C13—C8	42.0 (6)	C24—C23—C28—C27	−1.2 (8)
C18—C10—C14—C15	−72.9 (5)	C21—C23—C28—C27	177.1 (5)
C7—C10—C14—C15	170.0 (4)		
