# Supplementary figures and images for: Crystal structure of (E)-1-(4-chloro­phen­yl)ethanone O-de­hydro­abietyloxime
Source: Acta Crystallogr Sect E Struct Rep Online. 2014 Aug 1;70(Pt 9):o899–900. doi: 10.1107/S1600536814015888 (PMC4186074; doi:10.1107/S1600536814015888)

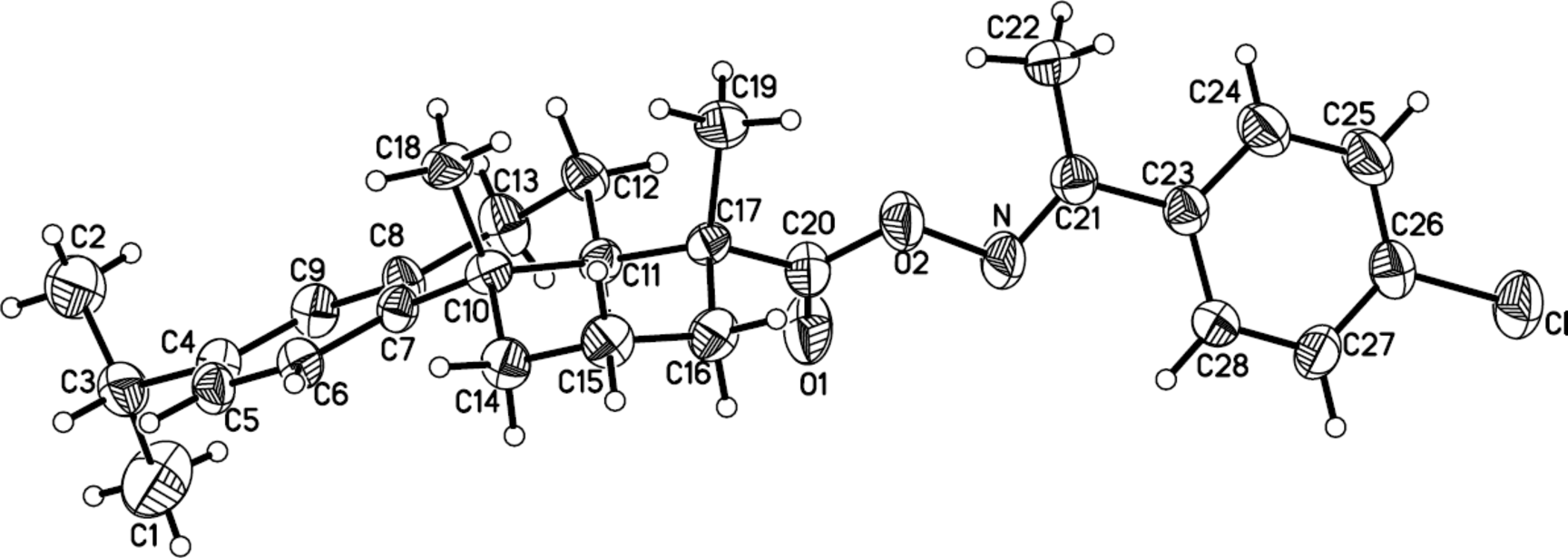

Supplement: Supplementary file 3 [file e-70-0o899-fig1.tif]
